# Yeast lifespan variation correlates with cell growth and *SIR2* expression

**DOI:** 10.1371/journal.pone.0200275

**Published:** 2018-07-06

**Authors:** Jessica T. Smith, Jill W. White, Huzefa Dungrawala, Hui Hua, Brandt L. Schneider

**Affiliations:** 1 Department of Cell Biology and Biochemistry, Texas Tech University Health Sciences Center, Lubbock, TX, United States of America; 2 Center for the Integration of STEM Education *&* Research, Texas Tech University, Lubbock, TX, United States of America; 3 Department of Biochemistry, Vanderbilt University School of Medicine, Nashville, TN, United States of America; 4 Department of Medical Education, Texas Tech University Health Sciences Center, Lubbock, TX, United States of America; Florida State University, UNITED STATES

## Abstract

Isogenic wild type yeast cells raised in controlled environments display a significant range of lifespan variation. Recent microfluidic studies suggest that differential growth or gene expression patterns may explain some of the heterogeneity of aging assays. Herein, we sought to complement this work by similarly examining a large set of replicative lifespan data from traditional plate assays. In so doing, we reproduced the finding that short-lived cells tend to arrest at senescence with a budded morphology. Further, we found that wild type cells born unusually small did not have an extended lifespan. However, large birth size and/or high inter-generational growth rates significantly correlated with a reduced lifespan. Finally, we found that *SIR2* expression levels correlated with lifespan and intergenerational growth. *SIR2* expression was significantly reduced in large cells and increased in small wild type cells. A moderate increase in *SIR2* expression correlated with reduced growth, decreased proliferation and increased lifespan in plate aging assays. We conclude that cellular growth rates and *SIR2* expression levels may contribute to lifespan variation in individual cells.

## Introduction

Life expectancy at birth is a statistical measure of the probability of the predicted lifespan for an average individual in a population. Within a population, lifespan can vary a great deal. The rate of aging may be a major factor in the variation of life expectancy. Numerous studies suggest that aging is impacted by genetic and environmental factors. In humans, genetic differences between individuals are estimated to contribute only 25–30% to the variation in life expectancy [1, 2]. Thus, environmental and other factors contribute substantially to the determination of lifespan [3]. However, considerable lifespan variation is also seen in populations of isogenic model organisms held in uniform and constant conditions [4]. Even the relatively simple budding yeast *Saccharomyces cerevisiae* demonstrates significant lifespan variation in individual cells [5–7].

Budding yeast, which asymmetrically divide to produce a limited amount of smaller and rejuvenated “daughter” bud cells, are an excellent tool for studying the progression of and mechanisms that contribute to aging [8]. The number of buds one yeast cell can produce, termed its replicative lifespan (RLS), is comparable to the aging of asymmetrically dividing higher eukaryote cells [9, 10]. Yeast aging research has produced noteworthy findings, including the discovery of the role of Sirtuins and the target of rapamycin (TOR) signaling pathway in modulating longevity [11, 12]. Additional studies have demonstrated the conservation of specific longevity genes and treatments between budding yeast and higher eukaryotes, supporting the idea that certain aspects of yeast aging research may be applicable to humans [10, 13]

Sir2 is an archetypal longevity gene and founder of the Sirtuin protein family. Sir2 is involved in the regulation of rDNA stability, the asymmetric retention of damaged proteins and less-fit mitochondria in mother cells, and the control of cellular lifespan [14]. While increased *SIR2* expression extends lifespan in yeast and other organisms [11], the factors and specific pathways that regulate *SIR2* expression are not well understood [15–18].

The pharmacological agent rapamycin slows growth and extends lifespan in yeast and a number of other model organisms [19]. The genetic targets of rapamycin, *TOR1* and *TOR2*, were first discovered in yeast [20]. The Tor signaling pathway modulates cell growth, cell size, and lifespan in response to nutrient availability [21]. In fact, deletion of a number of genes in the Tor signaling pathway reduces cell growth and cell size while increasing lifespan [12, 22, 23]. Similarly, glucose restriction (e.g. reduction from 2% to 0.05% also known as caloric restriction) and ibuprofen also reduce cell size and growth rate yet extend lifespan [24–26]. Yet a molecular mechanism connecting cell size or growth to lifespan has not been determined [27, 28]. Nonetheless, cell cycle control pathways impact longevity. Several studies have demonstrated that increased cell cycle times correlate with increased lifespan [24, 29]. In addition, deletion of key cell cycle regulators can have a profound impact on lifespan [29, 30]. Furthermore, Delaney et al. have shown that shorter-lived wild type cells displayed a budded phenotype at senescence suggesting that end-of-life cell cycle arrests contribute to lifespan variation [5]. Recently, Janssens and Veenhoff conducted an elegant study to examine the contribution of cell size and growth to the variation in individual cell lifespan [6]. Analysis of single wild type cells in a microfluidic lifespan assay revealed that excessive cell size increases may lead to the variation between individual cells [6]. For example, an early and rapid increase in cell size correlates with a shortened lifespan [6]. However, the potential role of cell size and/or growth rates on the variation of lifespan in wild type cells from traditional RLS aging assays has not been examined. Given the wealth of data produced from plate lifespan assays, the interplay between size, growth, and lifespan in a traditional plate assay is of great interest [10, 31, 32].

In this study, we investigated a large dataset of plate-based RLS assays in which birth size, death size, and cell growth rates were recorded. We reproduced the finding that cells with a budded morphology at senescence were short-lived. Further, we found that cells born unusually small did not have an extended lifespan. However, large birth size and/or high inter-generational growth rates significantly correlated with a reduced lifespan. Finally, we found that *SIR2* expression levels correlated with lifespan. *SIR2* expression was significantly reduced in large cells while increased *SIR2* expression correlated with decreased proliferation and growth. We conclude that cellular growth rates and *SIR2* expression levels may contribute to lifespan variation in individual cells.

## Materials & methods

### Replicative lifespan assays and size selection

The diploid BY4743 yeast strain and all knockout mutations were obtained from EUROSCARF. A *SIR2-*overexpression strain was kindly provided by Dr. Weiwei Dang as a haploid (YWD400) [33] and mated with BY4742 to create a diploid with one extra copy of *SIR2*. Wild type cells were transformed with either a low copy *SIR2* plasmid kindly provided by Dr. Danesh Moazed or a high copy *SIR2* plasmid created by cloning the *GPD1* promoter and wild type *SIR2* into the high copy pRS426 plasmid. Transformations were performed with the lithium acetate/SS carrier DNA/PEG method [34]. Cells were grown in standard yeast extract peptone dextrose (YPD) medium. Cells for centrifugal elutriation were grown in YPD for 3 days until the population was ~95% unbudded cells. Centrifugal elutriation was used for size fractionation as previously described [35]; eight fractions were collected in order of increasing mean cell volume. A sample was saved from the unelutriated, quiescent culture as a control. Liquid culture cell volumes and sizes were analyzed using a Z2 Coulter Counter Channelyzer and Accucomp software (v 3.01a). Aging assays were carried out on an MSM Series 300 micro-manipulator as previously described [36]. Mean lifespans were calculated from all cells that budded at least once.

### Photomicroscopy, measurement of cell size, and analysis

To determine mother and daughter birth size and budding size, cells were propagated on thin strips of YPD agar at 30°C in an incubation chamber on a Zeiss Axiovert 200 M microscope. Zeiss’s multidimensional acquisition program was used to follow 40–50 single cells during an 8–12 hour time course. An average of 3–4 cell cycles were observed for starting mother cells, and an average of 2–3 cell cycles were observed for the first generation of daughter cells. An AxioCamMR3 camera using EC Plan-Neofluar 40x objective (optovar 1.6) was used to capture images every 2 minutes. The Axiovision (v. 4.17) outline spline function was used to measure and determine cell diameters from the average of three independent measurements (data in S1 File).

A Nikon Coolpix 4500 and a 20x objective were used for the determination of cell size at the start and end of RLS assays of 917 wild type cells. Images were taken directly after birth of the cell, and then taken every 2–3 buds during the aging assay until death or senescence for 2 days. When cell “death” images were found to be significantly smaller than the previous image (due to post-death cell shrinkage), the picture and measurement were excluded from analysis. Birth and death images were imported into the Axiovision software and calibrated using an objective micrometer. The Axiovision outline spline function was used to manually measure and determine cell diameters from the average of three independent measurements. Cell volume was calculated from the determined cell diameters. To track cell intergenerational growth at the beginning of cell lifespan, 75 cells were randomly chosen from the 917 RLS cells dataset and sized at the 6^th^ and 10^th^ generation images. To track cells over their entire lifespan, a set of 20 cells with good picture quality for their entire lifespan were also randomly chosen from the 917 RLS cells dataset, and all of the images from the whole aging assay for those cells were sized (data in S1 File).

Statistical analysis of data was performed with Graphpad Instat (v3.10) and Graphpad Prism (v6.07). Unpaired t-tests, Welch corrected, were used to compare groups of cell lifespans, cell sizes, and AIGs. Mann-Whitney tests were used to analyze differences in lifespan curves. Visualization of the data was accomplished with Graphpad Prism (v6.07) and Excel 2013.

### RNA Isolation, real-time PCR and relative gene expression

Wild type BY4743 cultures were grown overnight at 30° in a tube rotator to ~2x10^7^ cells/ml in 5ml of YPD. Samples from centrifugal elutriation size selection were immediately used from elutriation. Cell size and concentration were measured with a Z2 Coulter Counter. Total RNA was harvested from wild type, control, and size-selected cells using a hot phenol method as previously described [37]. After checking the isolated RNA concentration and purity with a NanoDrop One^C^, the samples were diluted to 20 ng/ul. Real-time RT-PCR reactions were carried out using SuperScript III Platinum One-Step qRT-PCR Kit (Invitrogen) performed on a Bio-Rad CFX96 Touch System. TaqMan Gene Expression primer-probes for budding yeast *SIR2* and *ACT1* genes were obtained from Applied Biosystems. Gene expression analysis of the real-time data was conducted with Bio-Rad CFX Manager software as well as Excel 2013. Relative gene expression (data in S1 File) was calculated using the 2^-ΔΔ*C*^_T_ comparative *C*_T_ method as previously described [38].

## Results

### Yeast cells vary in lifespan and size

**Fig 1:**
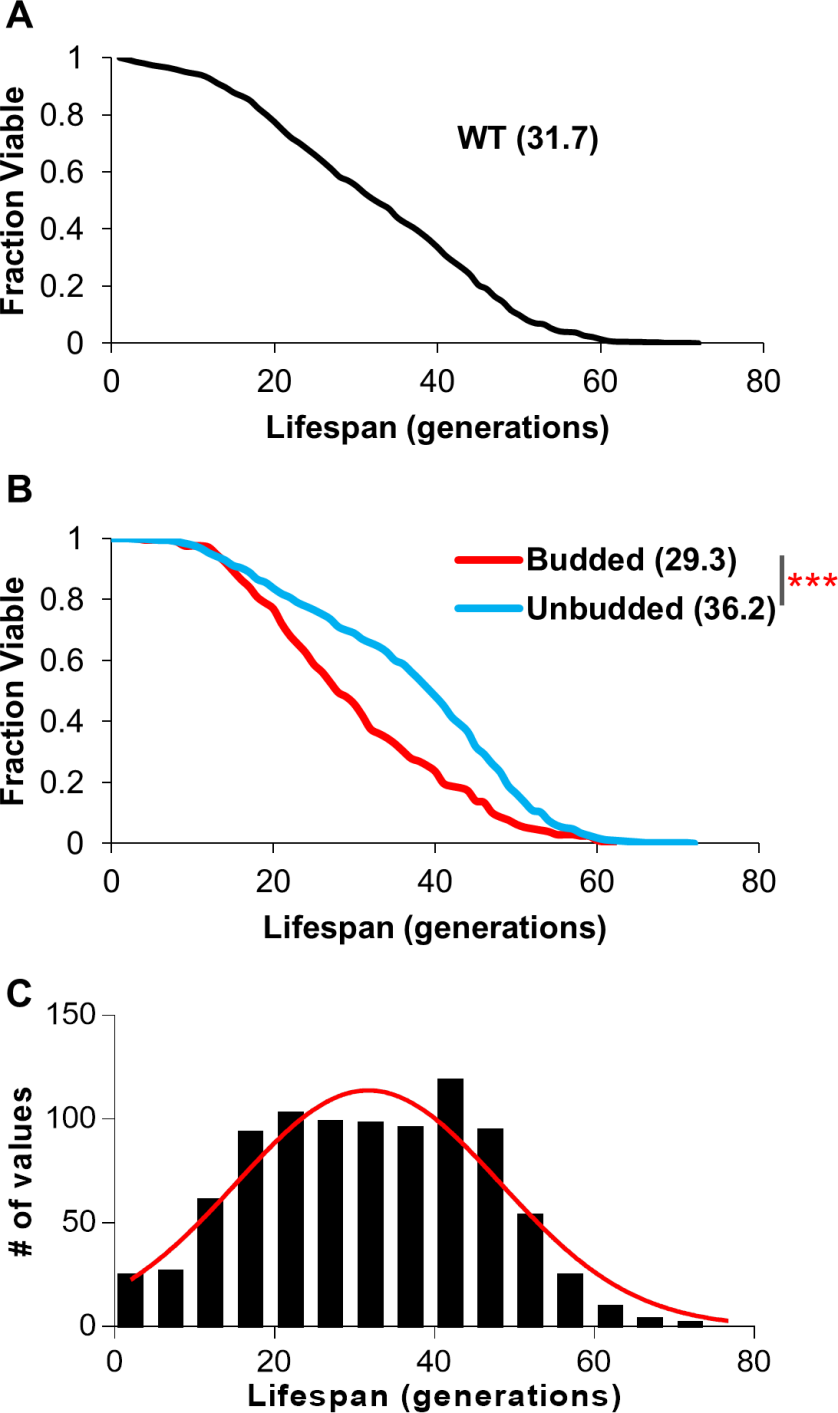
Wild type yeast cells display lifespan variation. (A) 917 diploid wild type BY4743 cells were aged on YPD plates and their lifespans were recorded. (B) End-of-life cell aging pictures were analyzed for budded (208 cells) or unbudded (508 cells) status. The lifespan curves of budded and unbudded cells are shown. (C) Lifespan values of the 917 cells were binned every 5 generations into a histogram, with a normal curve superimposed on the data to illustrate the relatively normal distribution. (*** = p<0.0001).

Janssens and Veenhoff recently examined lifespan variation in single wild type yeast cells using microfluidics and a data set of greater than 200 cells [6]. To complement this work, in this study, data was collected from 917 diploid wild type cells from traditional plate-based replicative lifespan assays. Plotting of survival curve data demonstrated the expected curve shape with an average RLS of 31.7 (Fig 1A), consistent with previous published reports [5, 30, 39]. Delaney et al. reported that under standard conditions, a significant proportion of cells in a lifespan assay display a budded end-of-life terminal morphology accompanied by a shorter lifespan [5]. Specifically, in a sample size of 529 BY4743 diploid cells at senescence, 78.4% were unbudded (35.6 RLS) and 21.6% were budded (26.6 RLS) [5]. Using the same strain, our data reproduced this finding with outcomes that were remarkably similar (Fig 1B). In our hands 70.1% were unbudded (36.2 RLS) while 29.9% were budded (29.3 RLS) (Fig 1B). Inherent variation in natural lifespan is not immediately apparent in survival curve plots. However, plotting survival data in histogram format reveals a roughly normal population distribution illustrating a wide range of variation (Fig 1C). The observed high degree of lifespan variation is consistent with observations recorded in other model organisms [4, 40, 41].

### Large cells are short-lived, but small cells are not long-lived

**Fig 2:**
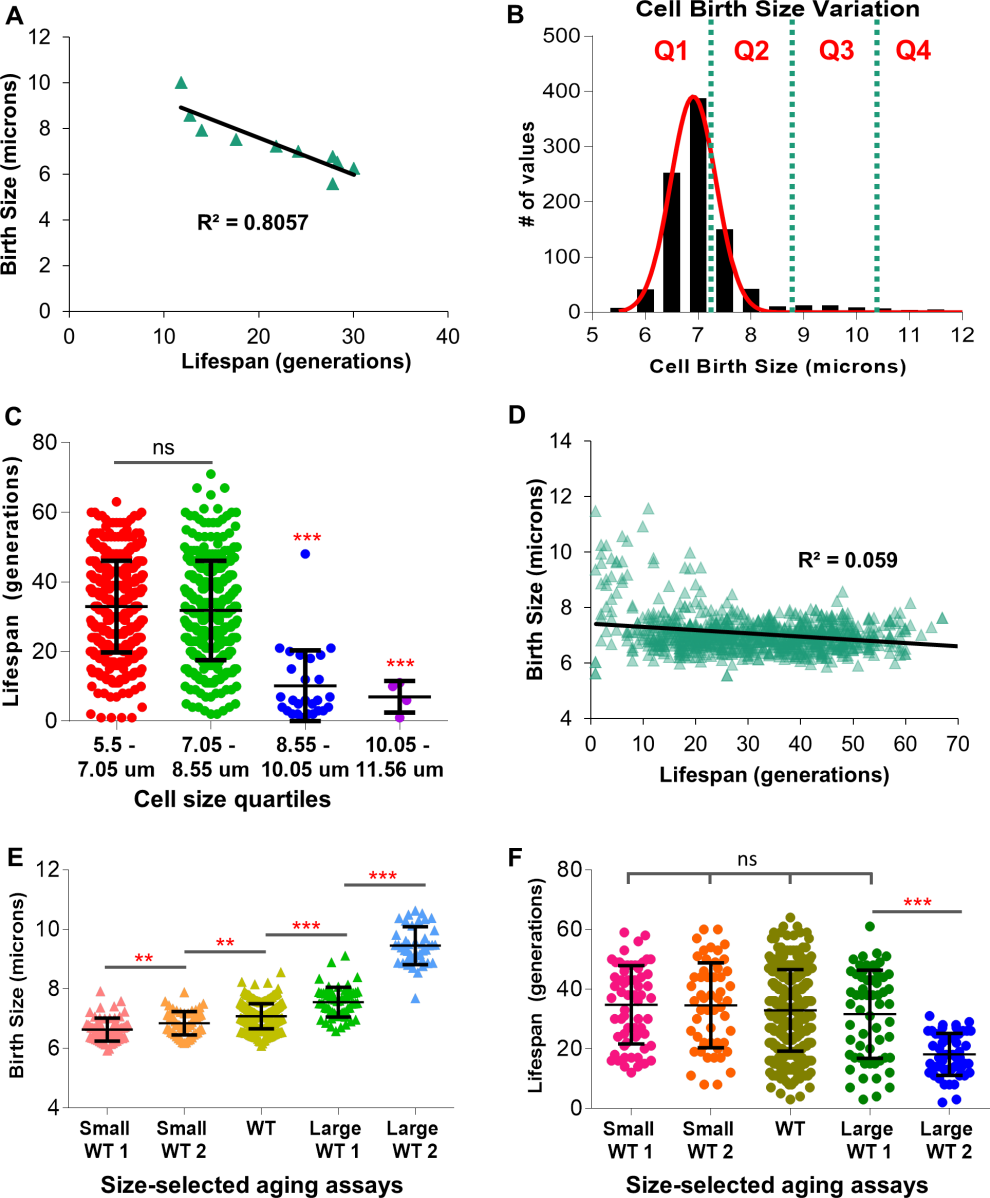
Wild type birth size correlates poorly with lifespan. (A) 2744 mutant and wild type cells were aged on YPD, DR, and YPD + treatment plates; their birth size, death size, and lifespan were recorded. Cell birth size deciles were calculated, and the decile birth size and lifespan were plotted. (B) A histogram of wild type birth sizes. Each bin is in 0.5 micron increments. The overlaid red line shows the relatively normal distribution. Overlaid dotted lines illustrate the division of birth size data into evenly divided quartiles (Q1-4). (C) Lifespan dot plots of the cells in each birth size quartile shown in 2B. (D) All 917 wild type cell birth sizes and lifespans (Pearson’s r = -0.2429). (E) Wild type cells were selected, via manual selection on the aging assay or via elutriation, for small or large cells at birth. Birth sizes of individual aging assays are displayed. (F) Lifespans of individual aging assays of wild type cells that were selected for small or large cells at birth. (** = p<0.001, *** = p<0.0001, ns = not significant).

Previous studies have shown that conditions or mutations that affect yeast cell size often also affect lifespan [24, 30, 31, 42, 43]. Examination of a very large data set of traditional plate aging studies (>2700 cells) performed in our lab demonstrated a correlation between birth size and lifespan (Fig 2A). Could variations in birth size of wild type cells be linked to lifespan variation in wild type cells? To examine the potential for a correlation between birth size and lifespan, a data set of 917 wild type cells were photographed and tracked from birth until death. A histogram plot of wild type birth size (Fig 2B) revealed a near normal distribution where the majority of cells range from 5.5 to 8.5 microns at birth. A small fraction of large cells can be visualized in the right most tail of the curve ranging in size from 8.5 to 11.5 microns (Fig 2B). Examination of the data set revealed that variation in birth size does not have a clear correlation with lifespan, except in the case of extremely large cells (Fig 2C). Dividing cells into equal quartiles based on birth diameter demonstrated that only the very largest cells at birth had a significantly reduced lifespan (Fig 2C and S1 Fig). Moreover, since the largest cells at birth are a small fraction of the total cells assayed (Fig 2B), the correlation between birth size and lifespan was weak (Fig 2D).

Independently, through the deliberate size selection of small or large cells, a potential correlation between birth size and lifespan was further examined. In confirmation of results reported above, the selection of cells statistically smaller at birth (Fig 2E) did not result in a population of cells with an extended lifespan (Fig 2F). Conversely, only cells with very large birth sizes (Fig 2E) were shown to have statistically shortened lifespans (Fig 2F). This is consistent with previous findings that daughters born from older mothers are frequently large and short-lived [44]. We conclude that, in the diploid BY4743 wild type strain, birth size variation has little correlation with population lifespan. The smallest-born cells in the population did not show a matching extension in lifespan, and birth size only has a negative correlation with lifespan in the largest-born cells in the population.

### Population cell size is modulated by growth to a critical cell size

**Fig 3:**
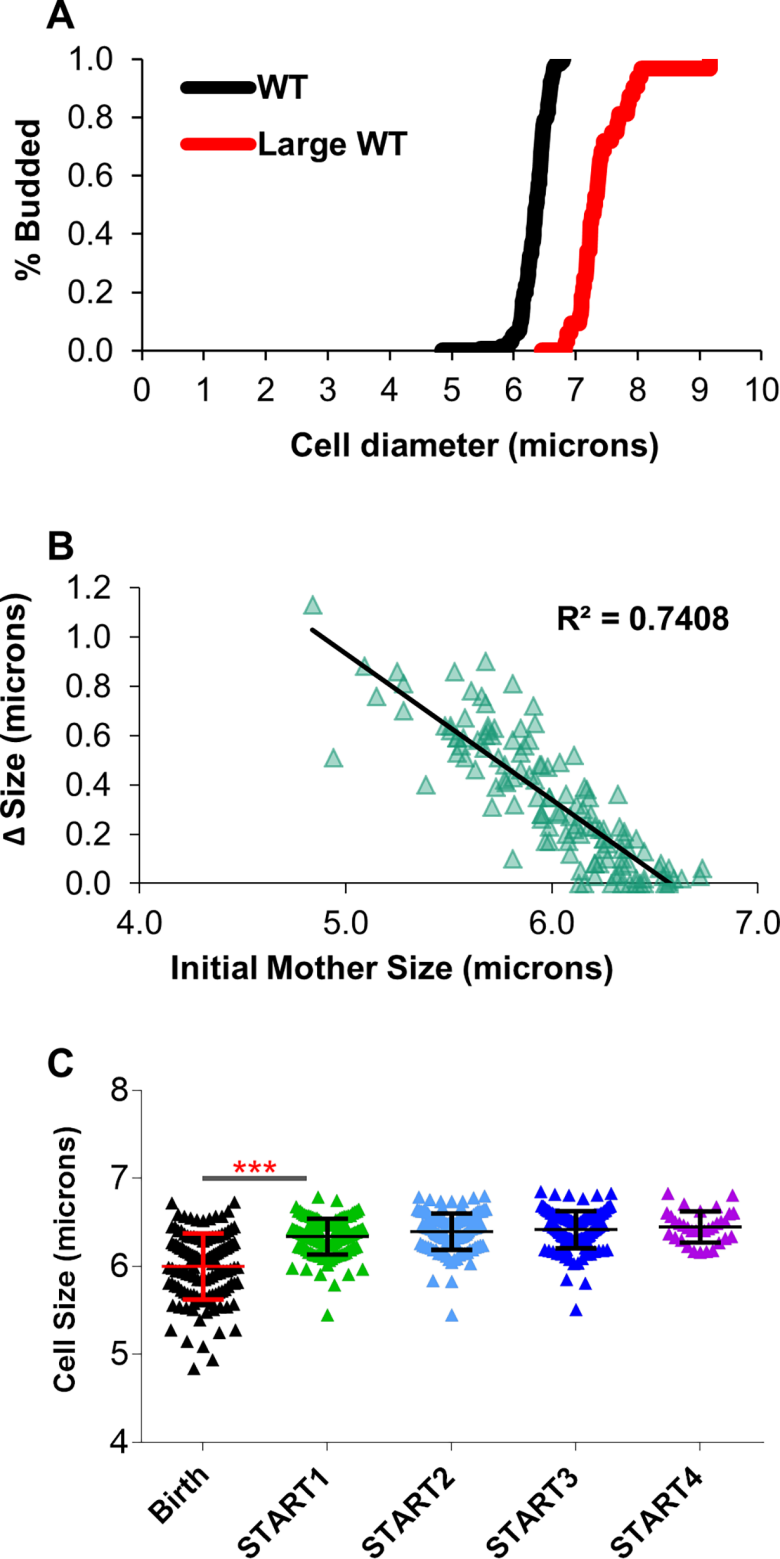
START reduces cell size variation. (A) 130 Wild type cells and a population of 32 large size-selected cells were imaged in a Zeiss Axiovert microscope. Initial mother cell size and cell size at first appearance of a bud were recorded. From the cell size data, the percentage of budded cells in the population vs. increasing cell size was calculated. Percent budded data for wild type cells, as well as the population of size-elutriated large wildtype cells was plotted. (B) 130 Wild type cells were imaged in a Zeiss Axiovert microscope. Initial mother cell size as well as the cell size at the appearance of a bud were recorded. The change in cell size over the G1 phase until first appearance of a bud was plotted against the initial cell size of mother cells. (C) Wild type cells were imaged for several cell cycles in a Zeiss Axiovert microscope. The variation in cell size of mother cells as they went through several cell cycles, starting with initial cell size, was plotted. (*** = p<0.0001, ns = not significant).

To examine why birth size appears to correlate with lifespan in cell size mutants but not wild type cells, the relationship between birth size and mean population size was examined. Does a lack of correlation between birth size and RLS reflect a significant increase in size between birth and initial budding? Specifically, do wild type cells born small grow to average size during the first cell cycle in an aging assay? To assess this, we measured initial birth size, and size at the first and subsequent buddings. While birth size can be variable (S2 Fig panel A), overall population cell size within a strain (S2 Fig panel B) is highly reproducible and maintained by cell size control mechanisms [22, 23, 45]. In fact, size homeostasis in yeast is predominantly achieved by linking cell cycle progression to the attainment of a minimum cell size (critical cell size for START, G1/S checkpoint) [45, 46]. Note that size variation observed at birth is reduced after one cell cycle (S2 Fig panel A). Importantly, critical cell size is specific to the genotype or the environmental conditions of the cell [25, 46, 47]. Thus, observations indicate that daughter cells must grow to the minimum cell size before they can progress past START, enter the cell cycle, and bud (Fig 3A). The black line in Fig 3A illustrates this point. Under standard aging conditions, diploid wild type cells must grow to ~6 microns in diameter to produce a bud. In contrast, daughter cells born larger than the critical cell size (red line, Fig 3A) bud almost instantly. Measurement of cell growth during the first cell cycle as a function of birth size confirms this observation (Fig 3B). Quantitation of the growth requirements for START revealed a close correlation between initial size and the amount of cell growth required for budding (Fig 3B). Specifically, small cells have a large growth requirement to be competent to bud, whereas in large cells the growth requirement is low (Fig 3B). A corollary of these results is that a size requirement for START should reduce size variability over time and normalize population cell size (S2 Fig panel A). Analysis of wild type cells during their first four buddings confirms this prediction (Fig 3C). Population size variability decreases after the first cell cycle and continues to decrease in subsequent cell cycles. Similar results are observed in mutants that produce either abnormally small (*rpl1b* or *rbl42a*) or large (*tif3* or *apn1*) cells (S3 Fig). In this case, small cell mutant cells are born small and the mean population stays small due to reduced critical cell size (S3 Fig panel A and B). The opposite is true for large cell size mutants (S3 Fig panel C and D). Thus, it is likely that critical cell size is the major determinant for mean population size (S3 Fig panel E). For example, mutants with a large critical cell size produce large daughter cells (e.g. ~7 microns for *apn1*) while the converse is for small cell mutants (e.g. ~5 microns for *rpl1b*) (S3 Fig panel A and D). Whereas regardless of birth size, wild type cells under standard aging conditions normalize to a size of ~6 microns (S2 Fig panel B). Thus, small birth sizes alone will not reduce population size unless the conditions that produced small daughters also reduce critical cell size. We conclude that population mean size is modulated by growth during the cell cycle, and that this growth can vary from cell to cell in a population. These results suggest that birth size does not correlate with RLS in wild type cells in part because small cells significantly increase in size during the first cell cycle.

### Does the rate of cell growth negatively correlate with lifespan?

**Fig 4:**
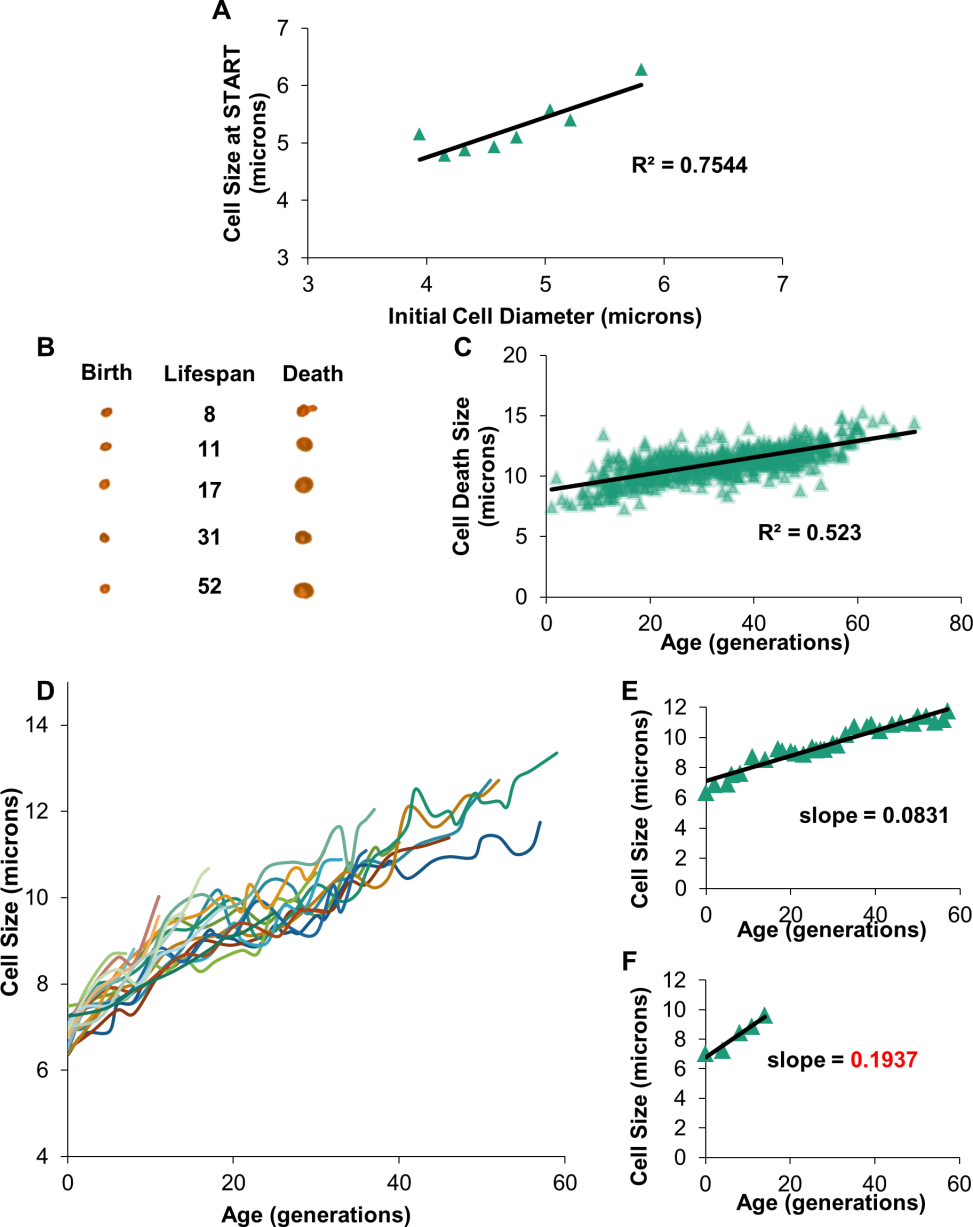
Intergenerational growth rate is variable. (A) Wild type cells were imaged in a Zeiss Axiovert microscope. Their initial size and the cell size at first budding are plotted. (B) 5 cells were randomly chosen from the dataset of 917 cells aged on plates; their birth and death pictures are displayed along with lifespan. (C) Cell death sizes for all 917 cells aged on plates were plotted against lifespan (Pearson’s r = 0.6395). (D) 20 cells were randomly chosen from the set of 917 cells to be sized in every picture taken over their entire lifespan. Their cell sizes were plotted against the age at which the pictures were taken. (E-F) Examples of the cells sized for their entire lifespan: one long-lived and one short-lived cell. Trendlines were fitted to the size data; the slope of the trendline is shown.

Previous studies have indicated that after the first cell cycle, yeast cells grow steadily over their lifespan [6, 9, 30, 48]. Examination of fractionated cells across a range of sizes reveals that within a population of cells the rate of cell growth correlates with cell size (Fig 4A), supporting observations that large cells grow proportionally faster than smaller cells [30, 49, 50]. By photographing individual wild type cells at birth and cell death in a standard aging assay, two observations were identified. First, death size was considerably larger and more variable compared to birth size (Fig 4B, S4 Fig). Second, the size at cell death positively correlated with lifespan (Fig 4C). This supports Janssens and Veenhoff’s conclusion that a maximal cell size does not limit lifespan [6].

The growth rate of individual cells was determined by photographing cells over the course of their lifespan. Analyses of these data revealed that while individual cells steadily increase in size, not all cells grow at the same rate (Fig 4D). Amongst a group of 20 randomly selected cells, a subset of cells increased in size at roughly double the rate of other cells (Fig 4E and 4F). In this case, the slope of the lines displayed mirrors the growth rate (Fig 4E and 4F). Previously, researchers have noticed a negative correlation between high rates of cell growth and lifespan [6, 30, 51], and observations presented here support a potential correlation between high growth rates and decreased lifespan (Fig 4D and 4E).

**Fig 5:**
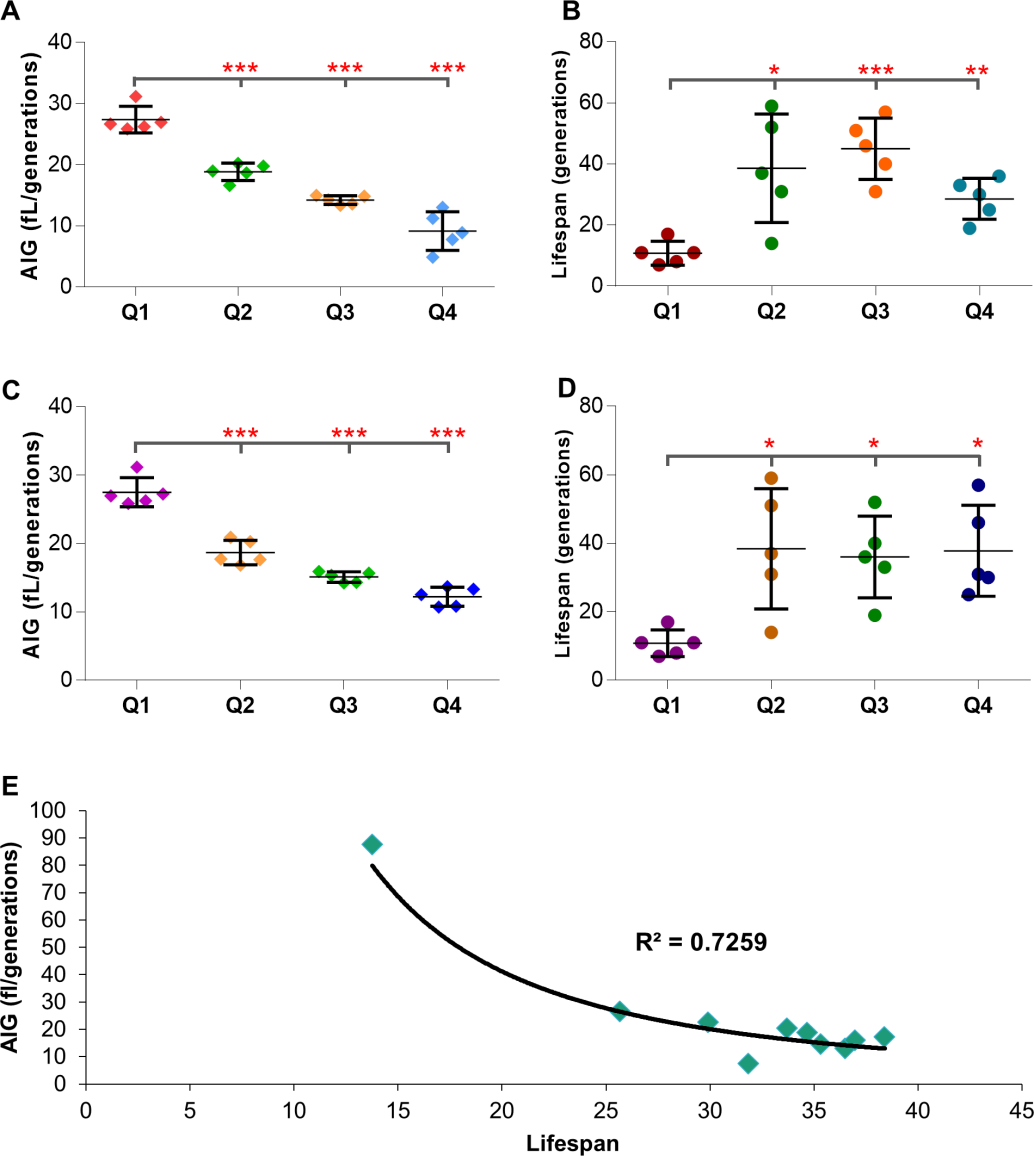
High intergenerational growth rates correlates with decreased lifespan. (A) Average intergenerational growth (AIG) of 20 cells up until a cutoff of bud 16, sorted into quartiles for highest and lowest AIG. (B) Lifespans of the sorted quartiles showing the variation in lifespan with higher and lower growth up until bud 16. (C) Average intergenerational growth (AIG) of 20 cells over their entire lifespan, sorted into quartiles for highest and lowest lifespan AIG. (D) Lifespans of the sorted quartiles showing the variation in lifespan of cells with higher or lower growth over total cell lifespan. (E) All 917 wild type cell total lifespan AIGs were sorted into deciles and plotted against lifespan. (* = p<0.05, ** = p<0.001, *** = p<0.0001).

Janssen et al. used photographed images of individual haploid wild type cells in a microfluidic aging assay to demonstrate that, even at age 5, there was a negative correlation between cell growth and lifespan [6]. We sought to determine if a similar negative correlation between cell growth and lifespan could be observed in young cells in the standard plate RLS assay. Using photographic measurements of individual cells and approximated volume measurements for a sphere, the average intergenerational growth (AIG), plotted as femtoliters (fL) / generation), can be calculated. Differences in the rate of cell growth as measured by AIG were easily observed even in the first few generations from birth to bud 6 (~1/5 of mean RLS) (S5 Fig panel A), but marked increases in early AIG did not translate to a negative correlation with eventual lifespan in this study (S5 Fig panel B). Since diploid cells are ~60% larger in volume and live 20–30% longer than haploid cells, we also examined if there was a negative correlation between cell growth and lifespan at age 10 (~1/3 of mean RLS) and 16 (~1/2 of mean RLS). Data obtained at age 10 was similar to age 6 (S5 Fig panel C and D). However, at age 16 a significant negative correlation between the rate of cell intergenerational growth AIG and lifespan was observed (Pearson’s r = -0.5282) (Fig 5A and 5B; S5 Fig panel E). Specifically, cells with the highest average intergenerational growth (Fig 5A) also had shorter lifespans (Fig 5B). Concurrently, the shortest-lived cells displayed the highest growth rates (Fig 4F, Fig 5A and 5B). In addition, this pattern could be extrapolated over an individual cell’s entire lifespan. In this case, the shortest-lived cells had a growth rate nearly twice as high as the average population (Fig 5C and 5D). Viewing the overall trends of average intergenerational growth for our entire dataset of 917 cells revealed that high rates of cell growth correlate with a shorter lifespan (Fig 5E, S6 Fig). We conclude that cell growth varies over the lifespan of individual cells and increased cell growth per generation (visible by the midpoint of a population’s lifespan) strongly negatively correlates with a shortened lifespan.

### Does *SIR2* gene expression correlate with cell size or growth rate?

**Fig 6:**
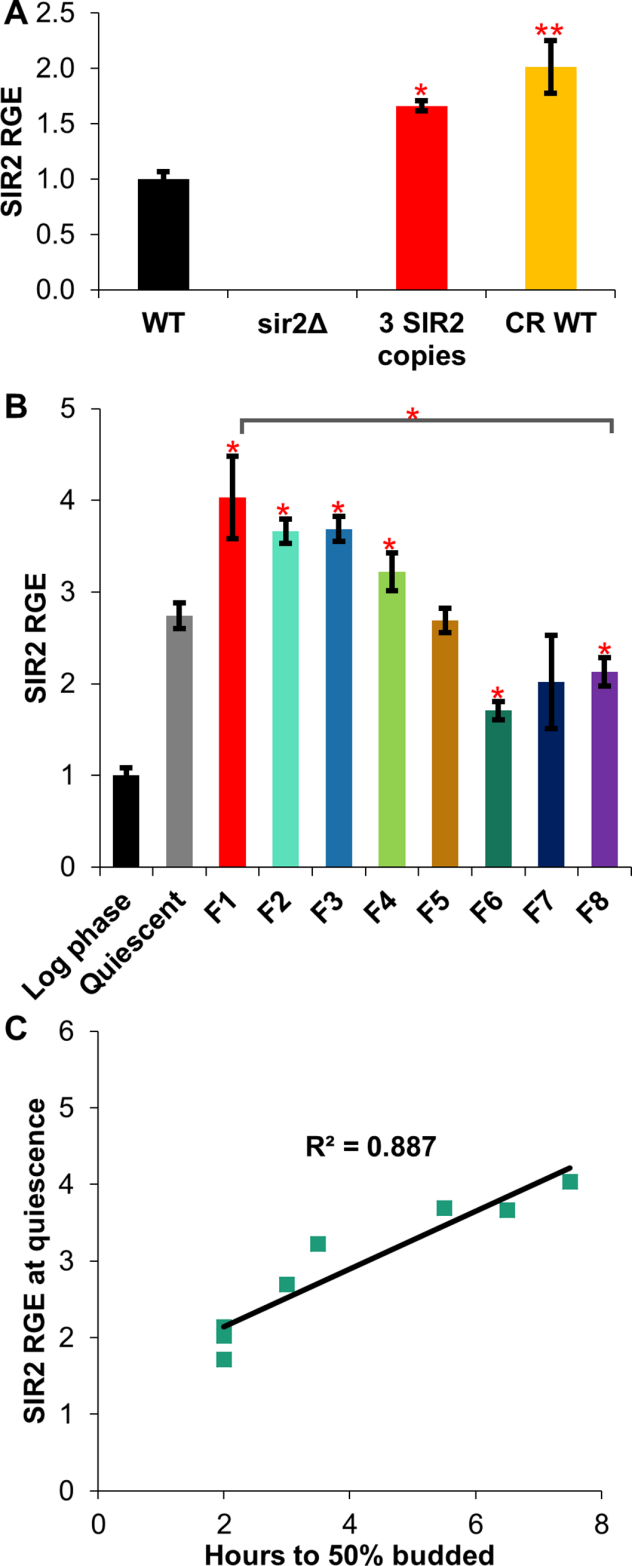
*SIR2* expression correlates with cell size and proliferation rates. (A) Relative gene expression (RGE) levels of *SIR2* in wild type, *sir2Δ*, wild type in CR, and overexpression of *SIR2* strains. (B) Relative gene expression levels of *SIR2* in size-fractionated cells. The unelutriated, quiescent control cells as well as a log phase culture are also included. The smallest elutriation fraction is F1 (33 fL), and the largest fraction is F8 (110 fL). A t-test measured the statistical difference of the size-fractionated elutriated cells from the non-elutriated T0 control. (C) Relative gene expression levels of *SIR2* in size-fractionated cells, (* = p<0.05, ** = p<0.001).

Diploid yeast cells vary in the amount of volume gained by each cell in a plate aging assay, even in the earliest generations. What could be the cause of such variation? Researchers have implicated the rDNA region and ribosomal biogenesis in the modulation of lifespan; poor nutrient conditions shift the rDNA region to a more inactive and silenced state, fewer rDNA genes are transcribed, and cell growth is reduced [52, 53]. The histone deacetylase gene *SIR2*, one of the most well-studied longevity genes, is an established regulator of the rDNA region [14]. Inactive or missing *SIR2* leads to reduced silencing and greater instability in the rDNA region, a reduction in lifespan, and an increase in cell size [14, 30, 54, 55]. In contrast, increased expression of *SIR2* is linked with a longer lifespan [11]. Despite these results, little is known about factors that influence *SIR2* expression. Therefore, we examined if *SIR2* expression was variable. In particular, potential relationships between *SIR2* expression, cell size, cell, growth, and cell cycle progression were investigated. Previous studies have shown that milder forms of caloric restriction (CR), such as 0.5% or 0.2% glucose, increase Sir2 protein levels [56]. However, potential changes to Sir2 expression in RLS-extending, more severe CR such as 0.05% glucose have not been investigated [57]. Using quantitative RT-PCR (qRT-PCR), our observations indicated that more severe CR moderately induces *SIR2* relative gene expression (RGE) to levels comparable to integrated *SIR2* expression constructs shown to increase lifespan (Fig 6A). Concomitantly, CR reduces birth size in plate aging assays and critical cell size (S7 Fig panel A and B). This fits with previous observations that CR reduces mean population cell size [25]. In addition, birth size was modestly increased in *sir2Δ* mutants on plate aging assays (S7 Fig panel A). To examine the potential for a link between *SIR2* expression and size, centrifugal elutriation was used to select eight differently sized fractions from a saturated, quiescent culture of wild type cells. These fractions ranged in mean cell volume from 33 fL (F1) to 110 fl (F8) (Fig 6B). Evaluation of *SIR2* expression in these fractions via qRT-PCR yielded several interesting results. First, *SIR2* RGE was significantly higher in the control quiescent cells compared to log phase cells (Fig 6B). Second, *SIR2* RGE was markedly higher in small cells compared to large cells (Fig 6B). For instance, *SIR2* RGE was nearly twice as high in the smallest cells compared to the largest cells (Fig 6B). The relationship between cell size and *SIR2* RGE became more profound when cell fractions were normalized for cell volume and the relative concentration per cell of *SIR2* expression was plotted (S7 Fig panel C). Investigation of a potential relationship between *SIR2* relative gene expression levels and cell cycle progression revealed a strong positive correlation (Fig 6C). Specifically, the cells with highest levels of *SIR2* gene expression had a G1-phase that was nearly four times as long as cells with the lowest levels of *SIR2* gene expression (Fig 6C).

**Fig 7:**
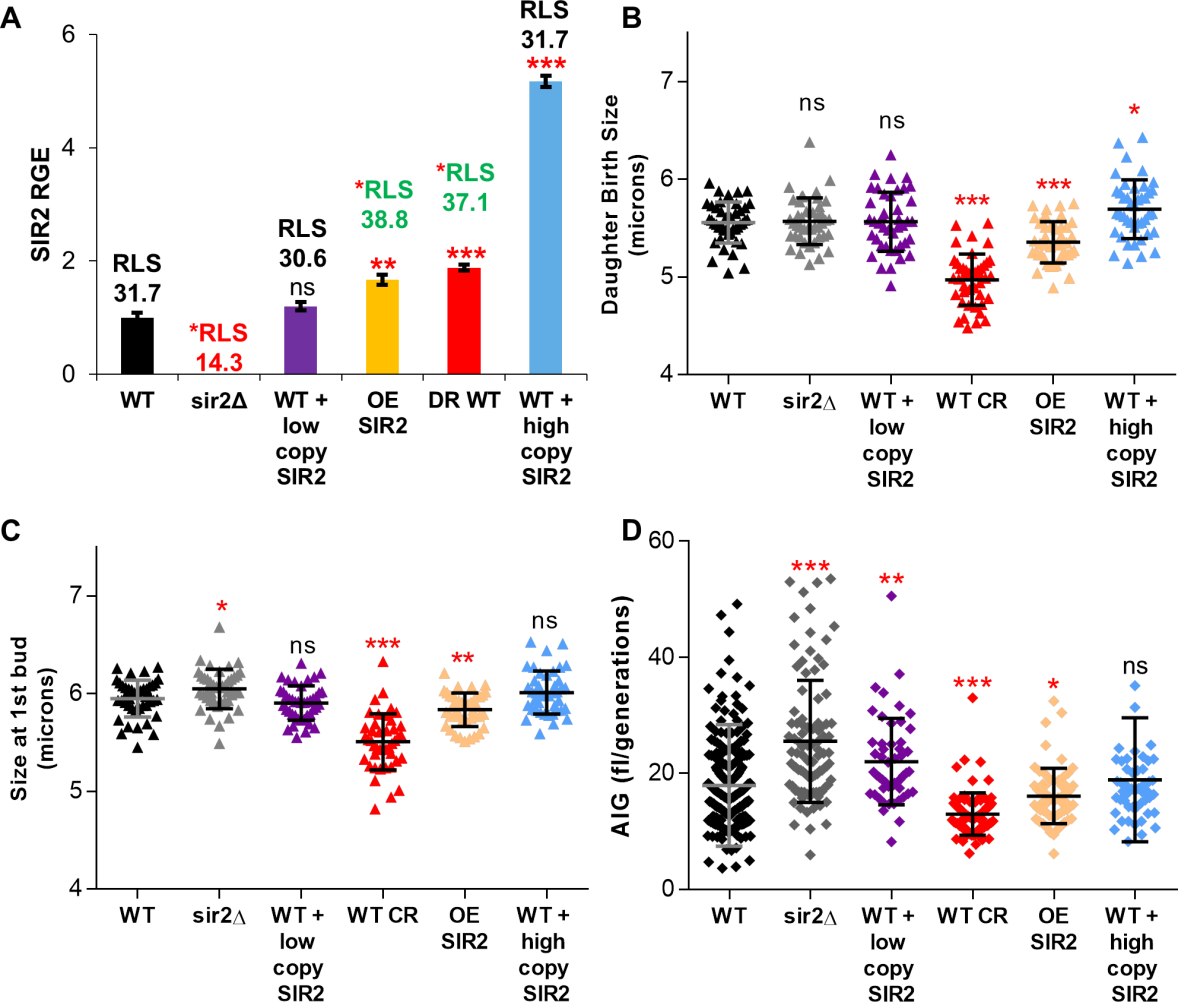
Modulation of *SIR2* expression affects cell size and intergenerational growth rates. (A) The relative gene expression levels of *SIR2* in wild type, *sir2Δ*, wild type transformed with a low copy *SIR2* plasmid, wild type in CR, overexpression of *SIR2* via an extra-integrated copy of *SIR2* (OE SIR2), and wild type transformed with a high copy *SIR2* plasmid strains is shown. Mean replicative lifespans are superimposed over the graph. Statistically significant differences in RLS and RGE levels are indicated. (B) Wild type, *sir2Δ*, wild type transformed with a low copy *SIR2* plasmid, wild type in CR, overexpression of *SIR2*, and wild type transformed with a high copy *SIR2* plasmid strains were imaged for several cell cycles in a Zeiss Axiovert microscope. The variation in birth size of cells immediately after separating from the mother was measured. (C) Wild type, *sir2Δ*, wild type transformed with a low copy *SIR2* plasmid, wild type in CR, overexpression of *SIR2*, and wild type transformed with a high copy *SIR2* plasmid strains were imaged for several cell cycles in a Zeiss Axiovert microscope. The variation in cell size at the appearance of the first bud was measured. (D) The average intergenerational growth (AIG) of wild type, *sir2Δ*, wild type transformed with a low copy *SIR2* plasmid, wild type in CR, overexpression of *SIR2*, and wild type transformed with a low copy *SIR2* plasmid strains is shown. (* = p<0.05, ** = p<0.001, *** = p<0.0001, ns = not significant).

To investigate the effect of *SIR2* expression levels on lifespan and growth, we utilized strains carrying either an integrated extra copy of *SIR2* (3 copies in a diploid cell), *SIR2* on a low copy plasmid, or *GPD-SIR2* on a high copy plasmid (Fig 7A). Interestingly, the strains with the highest and lowest *SIR2* expression did not display the lifespan extension (Fig 7A); only the strain with a modest increase in *SIR2* expression (equivalent to that observed in CR) displayed significant lifespan extension. In addition, a moderate increase in *SIR2* gene expression via CR medium or an extra copy of *SIR2* correlated with decreased cell size (Fig 7B) and decreased cell growth (Fig 7C and 7D, S8 Fig). In contrast, *sir2Δ* cells displayed increased cell growth, which may account for the observed increase in the cell size of *sir2Δ* cells (Fig 7C and 7D, S8 Fig). Conversely, wild type cells containing *SIR2* plasmids that did not increase lifespan exhibited small to no differences in birth size or initial rates of cell growth (Fig 7B and 7C, S8 Fig). Importantly, alterations in growth during the first cell cycle translated to differences in average intergenerational growth over the entire lifespan: strains with the highest lifespan extension also had the lowest average intergenerational growth (AIG) rates (Fig 7D). On the contrary, deletion of *SIR2* resulted in decreased lifespan and an increased AIG (Fig 7D). We conclude that *SIR2* expression, like cell growth per generation and lifespan, differs in a population of cells. *SIR2* expression varies with respect to cell size and is modestly elevated under conditions that promote longevity (e.g. CR). Finally, moderate induction of *SIR2* expression correlates with reduced intergenerational growth, slowed proliferation, and extended lifespan. These results suggest that variability in *SIR2* expression may contribute to the stochastic nature of replicative lifespan observed in wild type yeast and as such, mechanistic studies into the regulation of *SIR2* expression will be of great interest.

## Discussion

Countless studies have inferred that aging is inherent and unavoidable. Nonetheless, aging and factors that influence aging can have profoundly differing impacts on longevity. Clearly, intrinsic (e.g. genetic mutations) and extrinsic (environmental) factors have a role in lifespan variation; nonetheless, many questions remain [4–6]. In fact, dramatic variation of individual lifespans can be observed even in isogenic model organisms living in a nearly constant environment [6, 58–60]. This has led to the proposal that life expectancy is highly plastic and stochastic. The budding yeast is an ideal model organism for the discovery and elucidation of factors that contribute to the plasticity of life expectancy. Recently, Janssens and Veenhoff used microfluidics, a sensitive and quantitative approach, to examine potential sources for lifespan variation in wild type yeast cells [6]. We sought to complement this work by similarly examining a large set of replicative lifespan data from traditional plate assays. While the average RLS for the diploid wild type cells studied was 31.7, the lifespan of individual cells varied from 1–71 generations. The goal of this work was to examine potential sources for this variation. In so doing, our study surveys the role of intrinsic differences in cell size, growth rate, and *SIR2* gene expression levels on wild type lifespan variation. From this work, we observed: 1) Birth size correlates poorly with lifespan; 2) While high rates of intergenerational growth correlate with decreased lifespan, a maximal size is unlikely to limit lifespan; 3) *SIR2* gene expression is elevated in small cells and by CR, but reduced in large cells; and 4) Modest increases in *SIR2* expression (equivalent to that observed in CR) significantly increase lifespan and concomitantly decrease cell growth and cell size. In addition, we reproduced the finding that short-lived cells tend to arrest at senescence with a budded morphology [5]. We conclude that intrinsic differences in cellular growth rates and *SIR2* gene expression contribute some degree to the inherent variability of lifespan in yeast. The potential significance of these observations is discussed below.

### Is there a correlation between birth size and lifespan?

Our work and others have suggested that birth size might correlate with longevity [24, 30, 43, 51]. For example, numerous small cell mutants exhibit an extended lifespan while the converse can be true for large cell mutants (Fig 2A) [24, 30]. In contrast, others have suggested that the potential correlation between birth size and lifespan is weak and not universal [6, 24, 27, 28]. Thus, the first objective of this work was to determine if there was a correlation between the birth size of wild type cells and lifespan. While we could detect a nearly two-fold range in birth sizes, we observed no correlation between small birth size and a long-lived phenotype (Fig 2D). This remained true even when abnormally small cells were selected from the population (Fig 2E and 2F). Investigations of these observations revealed that a size requirement for START removed birth size variances by normalizing individual cells to the minimal size required for cell cycle progression (Fig 3). Thus, despite being born small, nearly all cells within a strain enter the first cell cycle at a nearly standardized cell size (Fig 3). A number of other studies have demonstrated that cells born small have a higher growth requirement for START as compared to cells born larger [24, 25, 47, 49]. Thus, we conclude that small birth size only correlates with a long-lived phenotype when the critical cell size is also reduced. For example, extrinsic factors like a reduction in the glucose levels from 2% to 0.05% extend lifespan when both birth size and critical cell size are reduced (Fig 7B and 7C; S7 Fig panel A and B). However, in the absence of external factors like CR or intrinsic cell size reducing mutations, we observed no correlation between reduced birth size and lifespan within a population of cells. In contrast, in cells born abnormally large, lifespan can be dramatically reduced. Since cells increase in size steadily as they age [9, 30], this could be simply due to the observation that daughters from older mothers tend to be born large and short-lived [44]. Conversely, it has been suggested that a maximal cell size may limit life expectancy [30, 61]. Yet, results reported here corroborate observations from Janssens and Veenhoff [6] that, in wild type cells, cell size at death is variable and a maximal cell size does not appear to limit lifespan.

### Increased intergenerational growth correlates with a reduced lifespan

Others and we have observed a potential correlation between cell growth and lifespan [6, 30, 51]. For example, nutrient sensing pathways play a large part in regulating the growth rate of cells as well as lifespan [12, 62–65]. In addition, ribosome biogenesis also modulates growth rate and lifespan [30, 39, 63, 66, 67]. Importantly, a considerable number of longevity mutants impact nutrient sensing pathways, ribosome biogenesis, or pathways involved in the control of protein translation [68]. Similarly, glucose restriction (0.05%) and other nutrient limitations reduce proliferation and growth rate (i.e. addition of cell mass) and concomitantly increase lifespan [25, 26, 69, 70]. In fact, several reports have linked a reduction in proliferation and/or growth rate to longevity and vice versa [6, 29, 30, 61, 67, 71]. Recently, when examining lifespan variation in wild type cells, Janssens and Veenhoff observed that high rates of cellular growth correlated with decreased lifespan [6]. By microscopically measuring the size of cells after each generation, we were able to observe individual cellular growth rates. From these observations, we confirmed that cells grow steadily between each budding. However, the growth rate is not constant for all cells. Some cells grow markedly faster than others (Fig 4). Cells that show the highest growth rate each generation (Figs 4D, 4F, 5A and 5C) are statistically the shortest-lived cells in the population (Fig 5B and 5D). While a difference in eventual lifespans based on growth rates cannot be detected for the earliest generations (S5 Fig), by the midpoint of an average diploid cell lifespan a statistical difference in cell fate can be visualized (Fig 5A and 5B). In concordance with Janssens & Veenhoff, we propose that high rates of intergenerational cell growth correlate with decreased lifespan. However, a potential mechanistic explanation for this observation remains elusive. Others and we have demonstrated that cell size often correlates with growth rates. Specifically, large cells frequently display high growth rates [30, 47, 50, 72]. Our finding that *SIR2* expression is low in large, rapidly growing cells (Fig 6B and 6C), as well as the finding that *SIR2* expression affects intergenerational growth (Fig 7D), may provide a potential mechanistic link between high rates of cell growth and decreased longevity, although much remains to be done in order to clarify the potential relationship. The physiological significance of these observations is considerably bolstered by the recent report that pancreatic acinar cell hypertrophy correlates with decreased lifespan across a broad range of organisms [73].

### A role for *SIR2* in lifespan variation?

**Fig 8:**
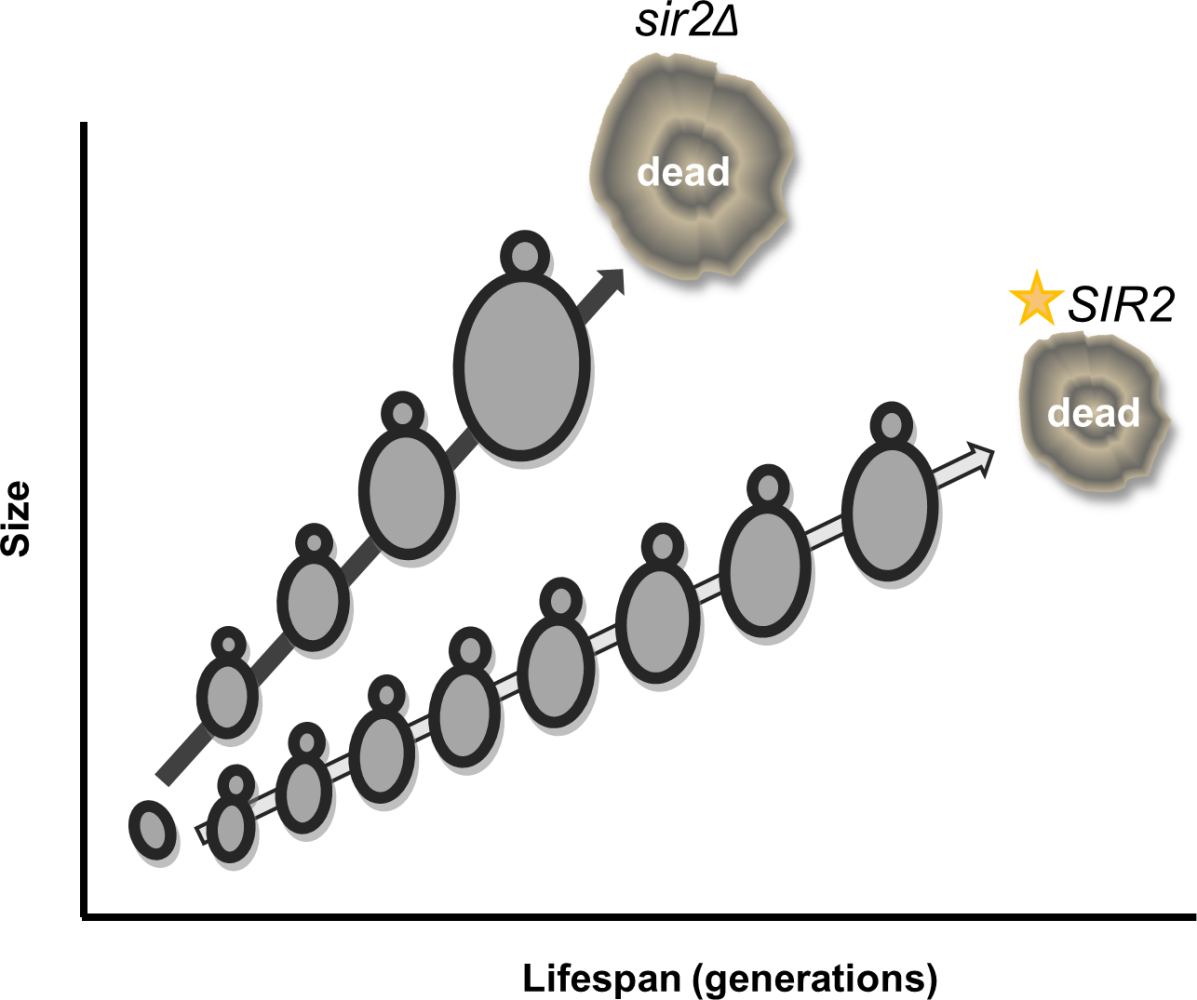
Working Model: Correlating growth rates and *SIR2* expression levels with longevity. Deletion of *SIR2* decreases lifespan and correlates with increased cell growth and cell size. In contrast, a modest increase in *SIR2* expression (*SIR2) achieved by either caloric restriction or an integrated additional copy of *SIR2* increases lifespan and correlates with decreased cell growth and cell size. While Sir2 appears to modulate cell size independently of known pathways [54], it is still intriguing to speculate that potential cross talk between genetic and environmental signals that influence growth rates and those that modulate *SIR2* expression could synergize to impact longevity.

Sir2, the archetypal anti-aging protein, is a NAD+ dependent histone deacetylase that regulates silencing at rDNA, telomeric, and mating-type loci [11]. *SIR2* expression levels appear to correlate with longevity. For example, moderate overexpression of *SIR2* extends lifespan, and vice versa [11]. Moreover, natural variations in *SIR2* expression correlate with strain longevity [74, 75]. Finally, Sir2 protein levels and Sir2-dependent silencing decrease with age [33]. An exciting new report demonstrated that heterochromatic rDNA regions undergo sporadic loss of silencing with increasing age [76]. Importantly, the authors show that intermittent waves of silencing at the rDNA loci are essential for longevity [76]. These results suggest that lifespan variation could reflect changes in the expression or activity of Sir2. Several labs have shown that increased Sir2 protein levels correlate with a variety of environmental stresses (i.e., oxidative stress, glucose restriction, or unfolded proteins) [56, 77, 78]. However, to date, little is known about the mechanisms that influence *SIR2* gene expression. Nutritional stress and ethanol have been shown to increase *SIR2* gene expression in *C*. *elegans* and yeast, respectively [77, 79]; while heat shock, a short rDNA region, and the Mig3 repressor are implicated in the down-regulation of *SIR2* [15, 18, 80]. Here, we report the novel finding that *SIR2* gene expression is increased in quiescent cells and by glucose restriction (Fig 6A). Since previous research demonstrates that Sir2 protein mirrors *SIR2* mRNA levels [81, 82], it is probable that the alterations in *SIR2* RGE reported here will translate into concomitant changes in Sir2 protein levels. Intriguingly, *SIR2* gene expression also appears to correlate with cell size and intergenerational growth. *SIR2* levels were dramatically higher in small compared to large cells (Fig 6B). The observed amount of *SIR2* expression in small and large cells in an elutriated population correlated with cell cycle time (Fig 6C), pointing to a possible relationship between *SIR2* levels and cell cycle progression. Furthermore, a moderate increase in *SIR2* expression correlates with slowed proliferation and intergenerational growth in traditional aging plate assays (Figs 6C, 7C and 7D), suggesting a potential feedback loop that may contribute to the stochastic nature of the replicative lifespan (Fig 8). Historically, an extreme increase in *SIR2* expression has been shown to affect viability and growth [83, 84], while a single extra copy extends lifespan [11]. The vast difference between intergenerational growth and lifespan responses to varying amounts of *SIR2* may result from the need for precise control of chromatin silencing [76], which could be disrupted by changes in *SIR2* gene copy or the addition of constitutive promoters (Fig 7A). *SIR2* expression may follow the Hormesis Theory of Aging: a beneficial response is seen at low levels of a stimulus or treatment, while further increased levels are harmful [85, 86]. A moderate increases in *SIR2* expression, either from genetic manipulation or inherent variation in expression, can result in decreases in intergenerational growth and increases in lifespan (Fig 7). Any changes to *SIR2* expression outside of the normal and beneficial levels may result in lifespan reduction, as the downstream effects of misregulated Sir2 deacetylation negatively affect normal cell processes [18, 84]. In addition, variation in the progressive loss of *SIR2* expression as cells grow and age [33] may randomly lead to a loss of silencing at rDNA and telomeric loci in a manner that ultimately becomes incompatible with viability [76].

## Supporting information

S1 Fig Decreased lifespan correlates with increased birth size. Cells were sorted by birth size into quartiles based on an even number of cells in each quartile. The lifespans of each quartile population are shown in the boxplot with the mean lifespan displayed in the middle of the boxplot. A t-test measured the difference in lifespans between each quartile. (* = p<0.05).
(TIF)

S2 Fig Wild type population size curves are reproducible despite varying birth size. (A) Wild type cells were imaged in a Zeiss Axiovert microscope. The variation in birth cell size and size at START was plotted. (B) 20 wild type population size curves are overlaid. (*** = p<0.0001).
(TIF)

S3 Fig Gene deletions shift birth size, size at START, and population size curves. Diploid deletion mutant cells were imaged for several cell cycles in a Zeiss Axiovert microscope. The variation in cell size of small cell mutants (*rpl1b*Δ and *rpl42a*Δ) (A-B) and large cell mutants (*tif3*Δ and *apn1*Δ) (C-D) from birth size through three consecutive cycles (START 1–3) was plotted. (E) The Z2 Coulter Counter size curves for the *rpl1b*Δ and *apn1*Δ mutants are shown. (** = p<0.001, *** = p<0.0001, ns = not significant).
(TIF)

S4 Fig Death size varies more than birth size. The birth and death size of 917 wild type cells are shown in a dot plot. The variation of birth and death size was evaluated using the f test. (*** = p<0.0001).
(TIF)

S5 Fig Early intergenerational growth rates correlate poorly with lifespan. (A) Cells were sorted by average intergenerational growth rates (AIG) from birth to bud 6 into quartiles based on an even number of cells in each quartile. (B) The lifespans of the quartiles, illustrating that growth from birth to bud 6 does not correlate with a change in ultimate lifespan. (C) Cells were sorted by AIG from birth to bud 10 into quartiles based on an even number of cells in each quartile. (D) The lifespans of the quartiles, illustrating that growth from birth to bud 10 does not correlate with a change in ultimate lifespan. (E) AIG from birth to bud 16 negatively correlates with lifespan (Pearson’s r = -0.5282). (F) Cells that grow more than the median AIG up to bud 16 live shorter lives, and vice versa. (* = p<0.05, ns = not significant).
(TIF)

S6 Fig Intergenerational growth rates over the whole lifespan correlate with cell lifespan. (A) Cells were sorted by lifespan into evenly numbered quartiles. The volume increase per generation for every budding event was calculated for each cell. The shortest-lived cells were most likely to have higher AIGs for every division. (B) All 917 cell AIGs were plotted against cell lifespan (Pearson’s r = -0.2004). (* = p<0.05, ns = not significant).
(TIF)

S7 Fig Both birth size and critical cell size are reduced in CR. (A) Wild type, *sir2Δ*, wild type with an extra copy of *SIR2* (OE SIR2), and wild type in CR virgin daughter cells were aged on traditional aging plates. Birth sizes of the virgin daughter cells at the beginning of the aging assay were recorded. (B) Wild type cells were imaged in a Zeiss Axiovert microscope in both YPD (2% glucose) and CR (0.05% glucose) media. Birth size and size at appearance of first bud (critical size) were recorded. (C) Relative gene expression levels of *SIR2* in size-fractionated cells, normalized by the mean cell volume of each fraction. The unelutriated, quiescent control cells as well as a log phase culture are also included. The smallest fraction is F1, and the largest fraction is F8. A t-test measured the statistical difference of the size-fractionated elutriated cells from the non-elutriated T0 control. (* = p<0.05, ** = p<0.001, *** = p<0.0001, ns = not significant).
(TIF)

S8 Fig Intergenerational growth is affected by altering *SIR2* expression levels. Wild type, *sir2Δ*, wild type transformed with a low copy *SIR2* plasmid, wild type in CR, overexpression of *SIR2* via an extra integrated copy of *SIR2* (OE SIR2), and wild type transformed with a high copy *SIR2* plasmid strains were imaged for several cell cycles in a Zeiss Axiovert microscope. The size of cells upon appearance of the second bud was measured. (** = p<0.001, *** = p<0.0001).
(TIF)

S1 File Data on cell sizes, volumes, intergenerational growth, budded status at death, lifespan, and relative gene expression. Datasets for all figures in the paper. Each sheet corresponds to a figure in respective order listed in the paper.
(XLSX)
