# Supplementary figures and images for: Yeast lifespan variation correlates with cell growth and SIR2 expression
Source: PLoS One. 2018 Jul 6;13(7):e0200275. doi: 10.1371/journal.pone.0200275 (PMC6034835; doi:10.1371/journal.pone.0200275)

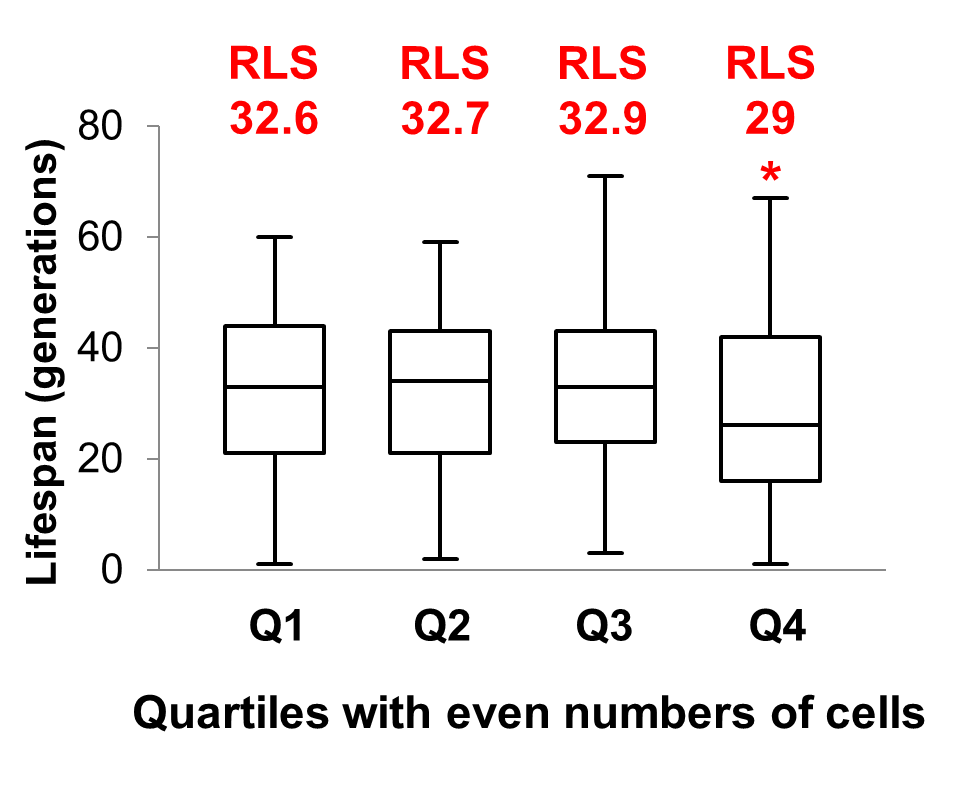

Supplement: S1 Fig — Cells were sorted by birth size into quartiles based on an even number of cells in each quartile. The lifespans of each quartile population are shown in the boxplot with the mean lifespan displayed in the middle of the boxplot. A t-test measured the difference in lifespans between each quartile. (* = p<0.05). (TIF) [file pone.0200275.s001.TIF]

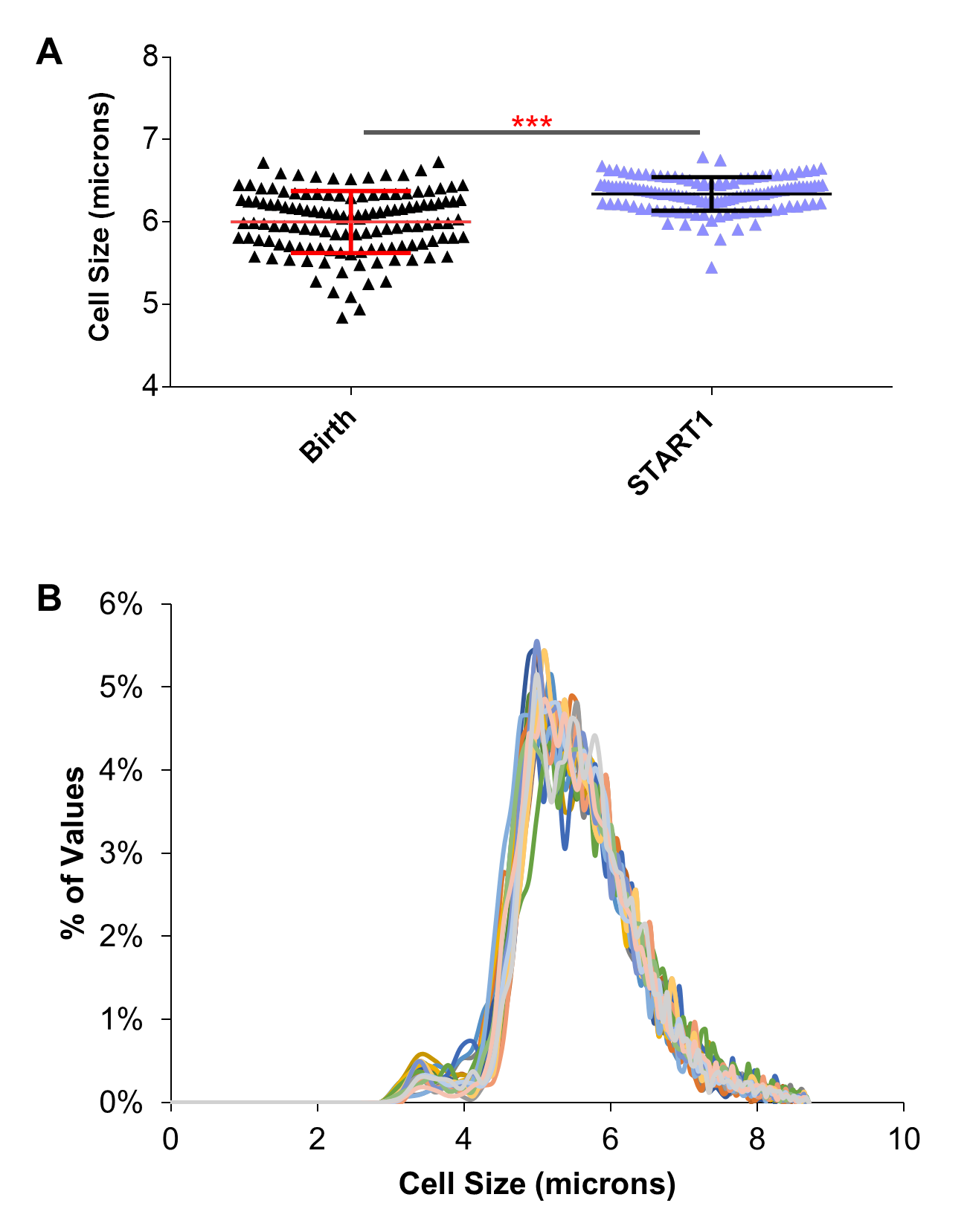

Supplement: S2 Fig — (A) Wild type cells were imaged in a Zeiss Axiovert microscope. The variation in birth cell size and size at START was plotted. (B) 20 wild type population size curves are overlaid. (*** = p<0.0001). (TIF) [file pone.0200275.s002.TIF]

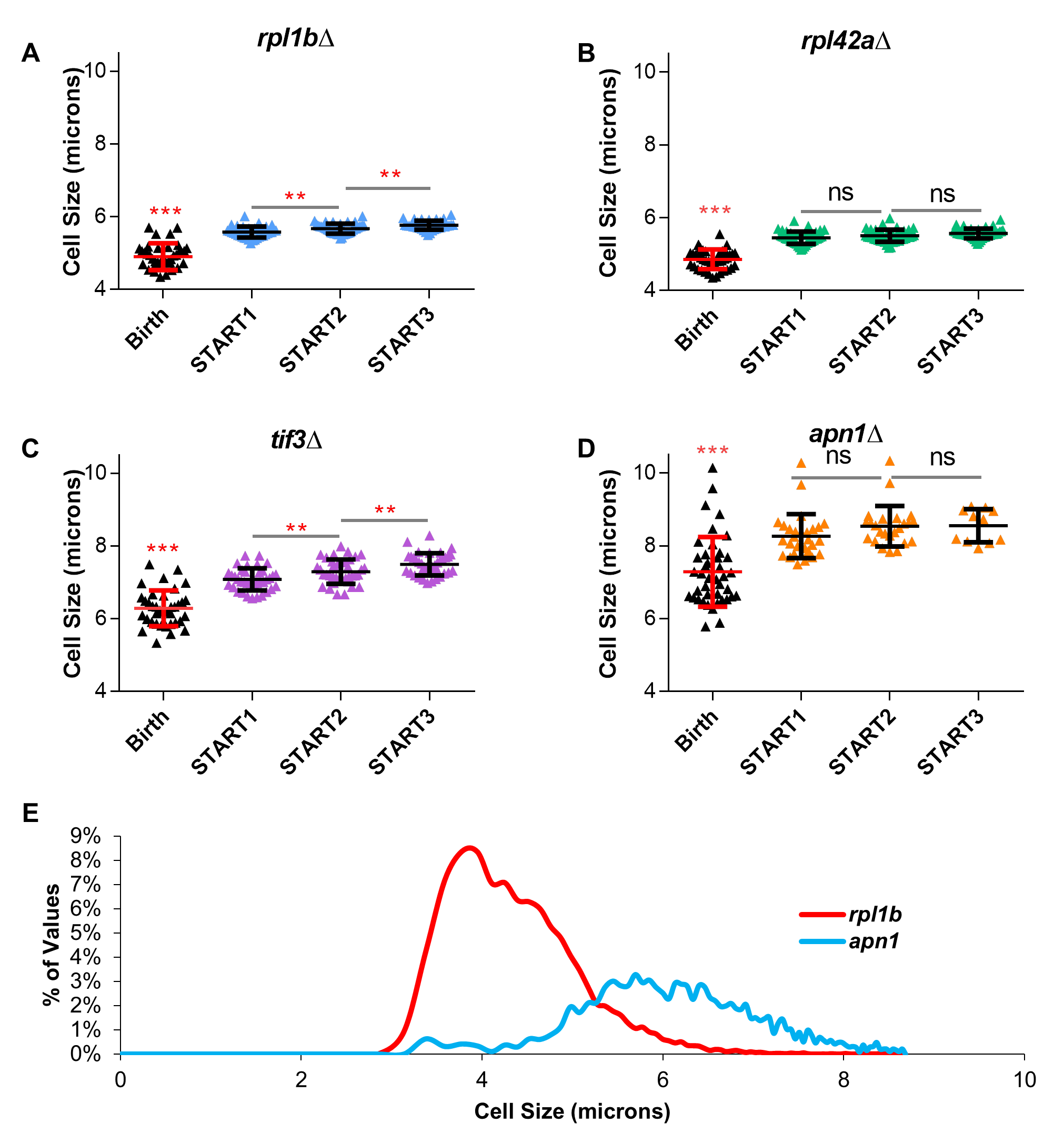

Supplement: S3 Fig — Diploid deletion mutant cells were imaged for several cell cycles in a Zeiss Axiovert microscope. The variation in cell size of small cell mutants (rpl1bΔ and rpl42aΔ) (A-B) and large cell mutants (tif3Δ and apn1Δ) (C-D) from birth size through three consecutive cycles (START 1–3) was plotted. (E) The Z2 Coulter Counter size curves for the rpl1bΔ and apn1Δ mutants are shown. (** = p<0.001, *** = p<0.0001, ns = not significant). (TIF) [file pone.0200275.s003.TIF]

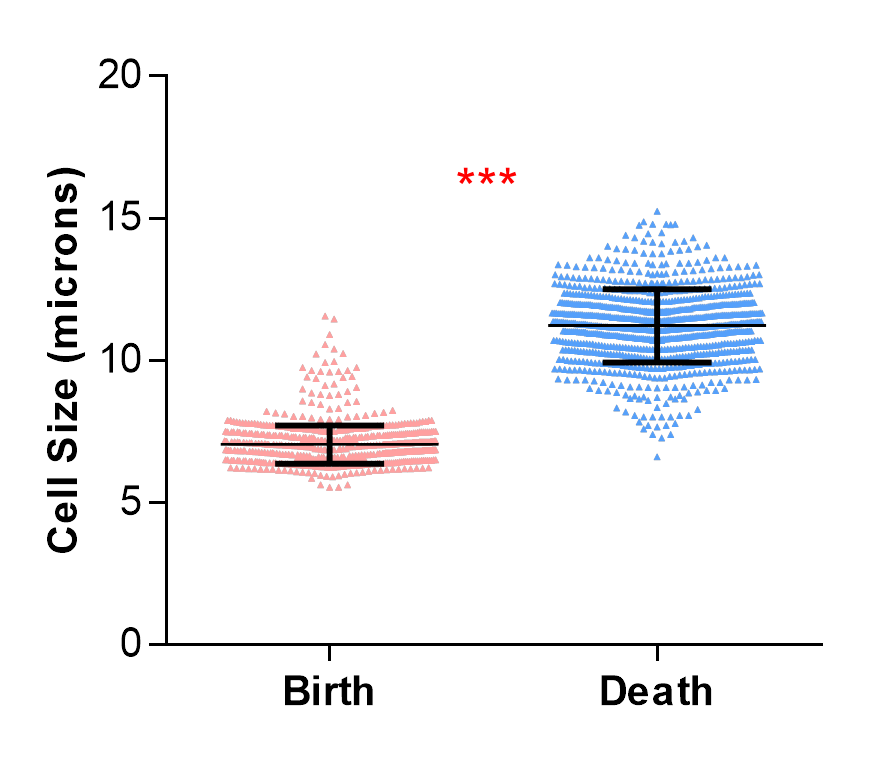

Supplement: S4 Fig — The birth and death size of 917 wild type cells are shown in a dot plot. The variation of birth and death size was evaluated using the f test. (*** = p<0.0001). (TIF) [file pone.0200275.s004.TIF]

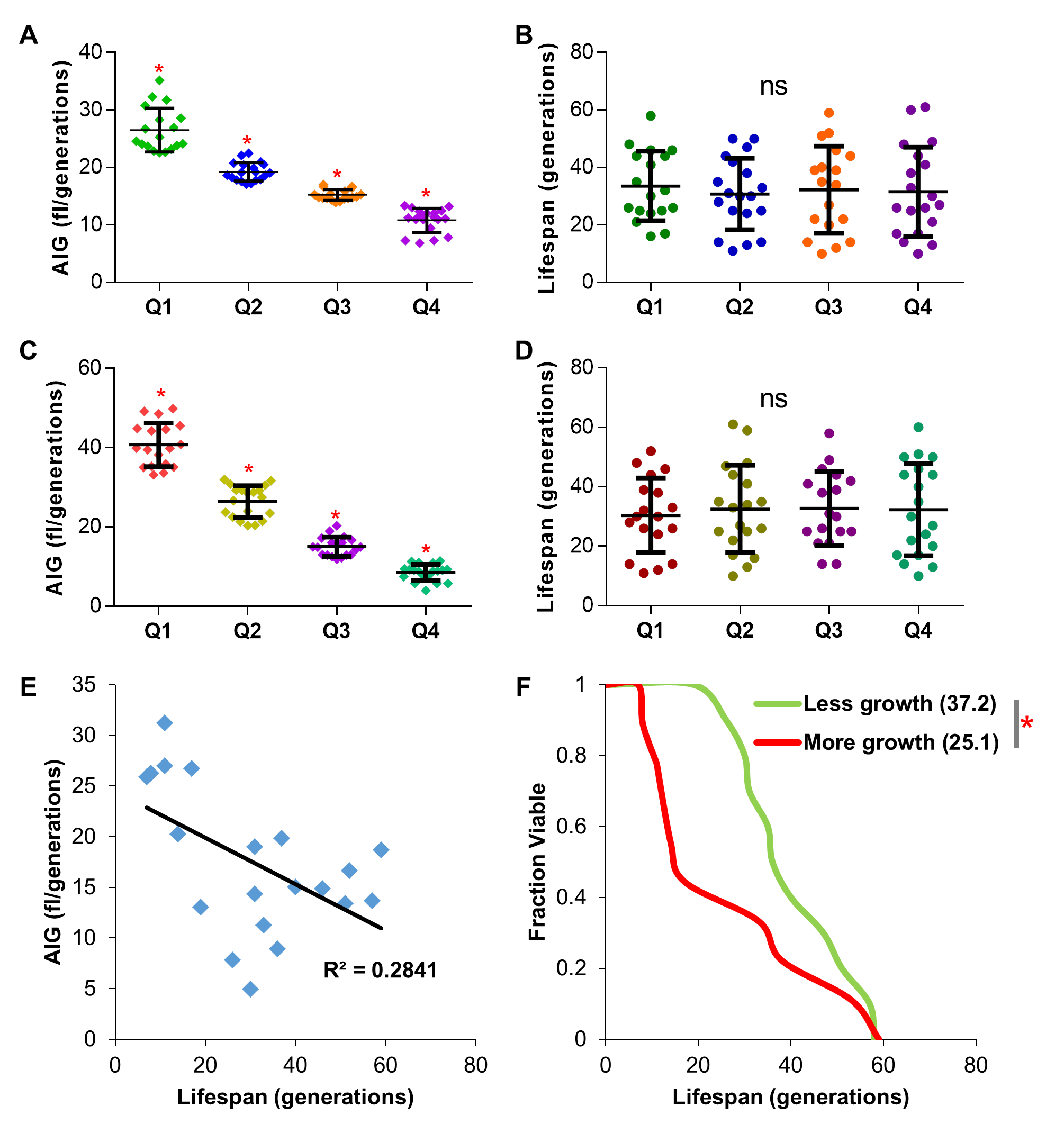

Supplement: S5 Fig — (A) Cells were sorted by average intergenerational growth rates (AIG) from birth to bud 6 into quartiles based on an even number of cells in each quartile. (B) The lifespans of the quartiles, illustrating that growth from birth to bud 6 does not correlate with a change in ultimate lifespan. (C) Cells were sorted by AIG from birth to bud 10 into quartiles based on an even number of cells in each quartile. (D) The lifespans of the quartiles, illustrating that growth from birth to bud 10 does not correlate with a change in ultimate lifespan. (E) AIG from birth to bud 16 negatively correlates with lifespan (Pearson’s r = -0.5282). (F) Cells that grow more than the median AIG up to bud 16 live shorter lives, and vice versa. (* = p<0.05, ns = not significant). (TIF) [file pone.0200275.s005.TIF]

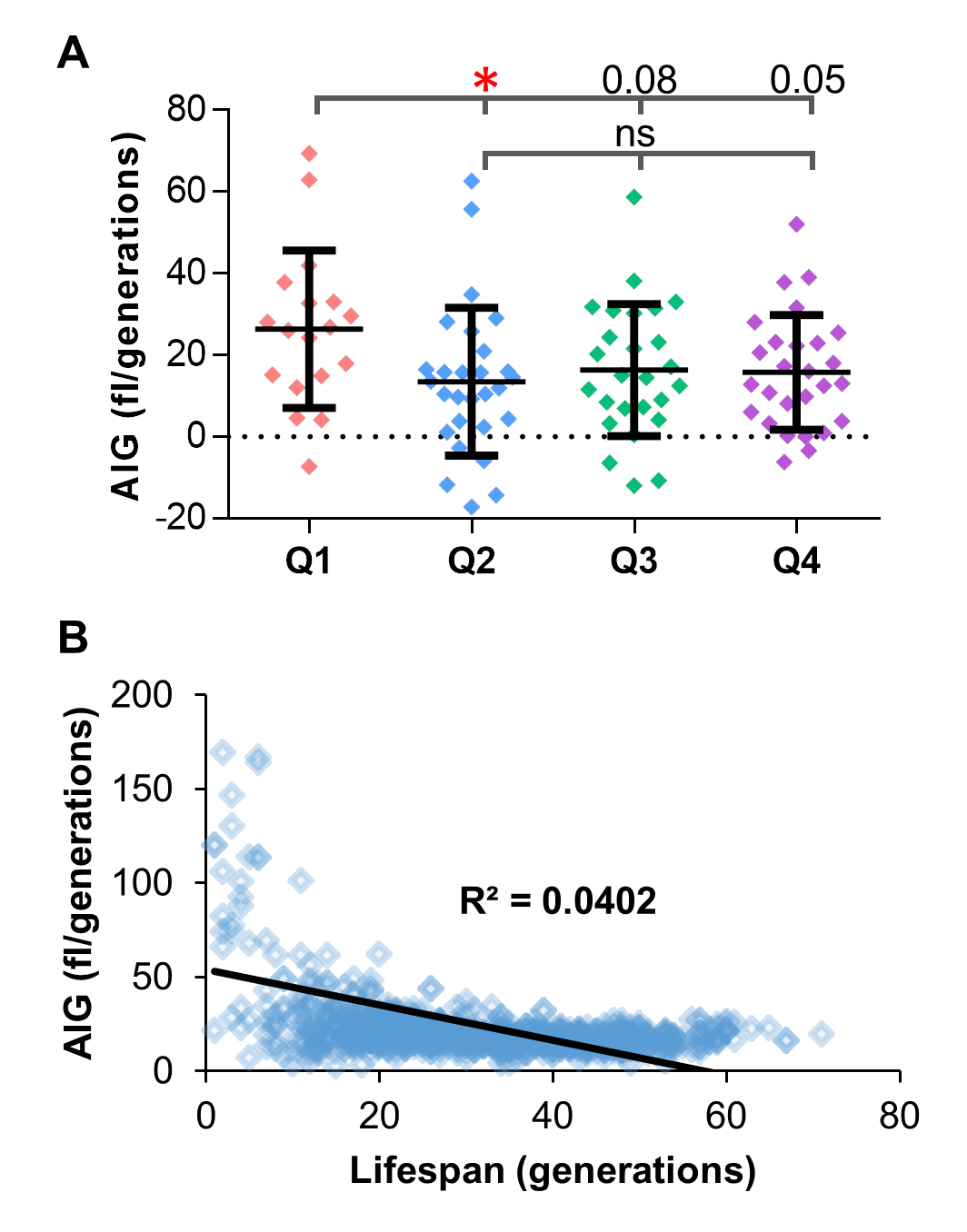

Supplement: S6 Fig — (A) Cells were sorted by lifespan into evenly numbered quartiles. The volume increase per generation for every budding event was calculated for each cell. The shortest-lived cells were most likely to have higher AIGs for every division. (B) All 917 cell AIGs were plotted against cell lifespan (Pearson’s r = -0.2004). (* = p<0.05, ns = not significant). (TIF) [file pone.0200275.s006.TIF]

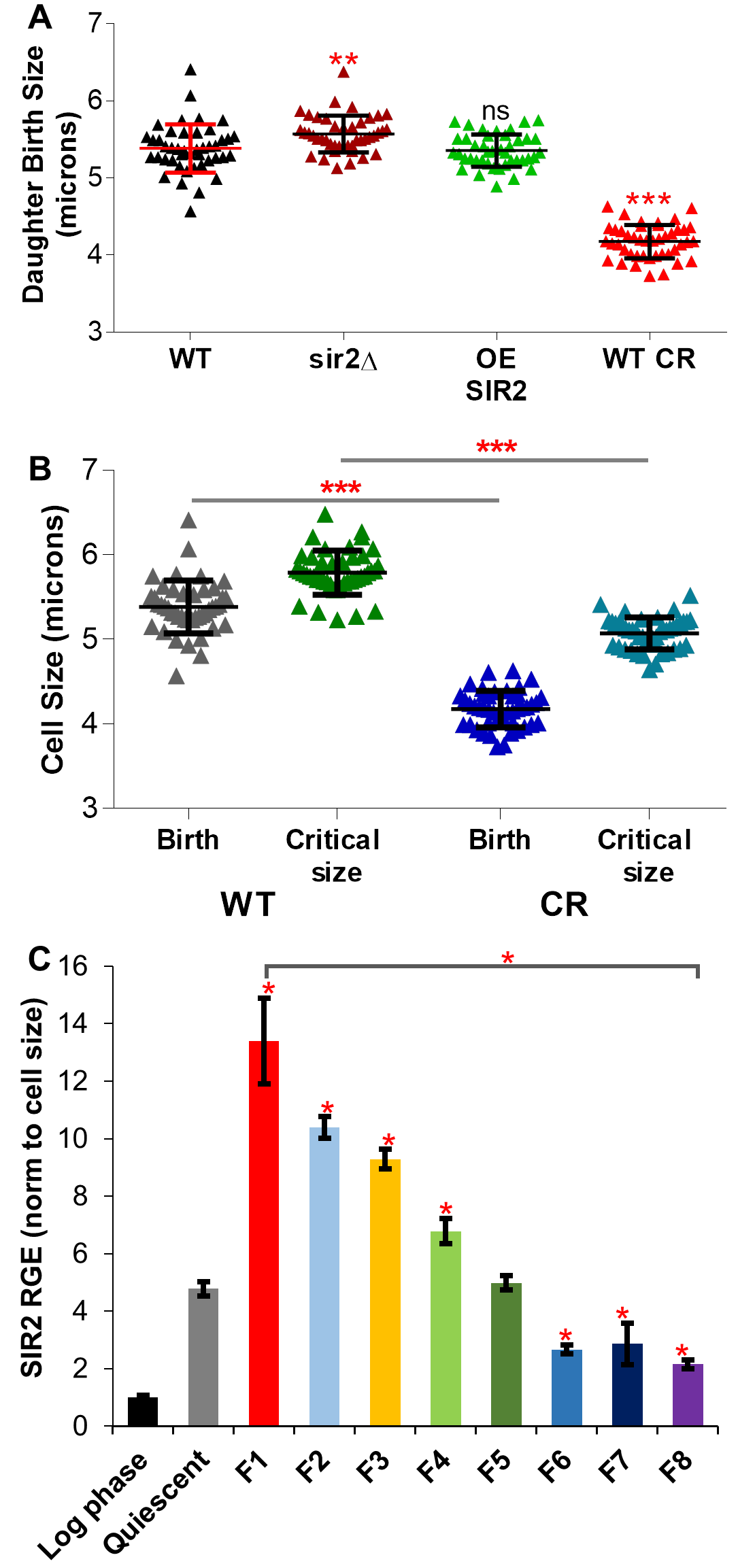

Supplement: S7 Fig — (A) Wild type, sir2Δ, wild type with an extra copy of SIR2 (OE SIR2), and wild type in CR virgin daughter cells were aged on traditional aging plates. Birth sizes of the virgin daughter cells at the beginning of the aging assay were recorded. (B) Wild type cells were imaged in a Zeiss Axiovert microscope in both YPD (2% glucose) and CR (0.05% glucose) media. Birth size and size at appearance of first bud (critical size) were recorded. (C) Relative gene expression levels of SIR2 in size-fractionated cells, normalized by the mean cell volume of each fraction. The unelutriated, quiescent control cells as well as a log phase culture are also included. The smallest fraction is F1, and the largest fraction is F8. A t-test measured the statistical difference of the size-fractionated elutriated cells from the non-elutriated T0 control. (* = p<0.05, ** = p<0.001, *** = p<0.0001, ns = not significant). (TIF) [file pone.0200275.s007.TIF]

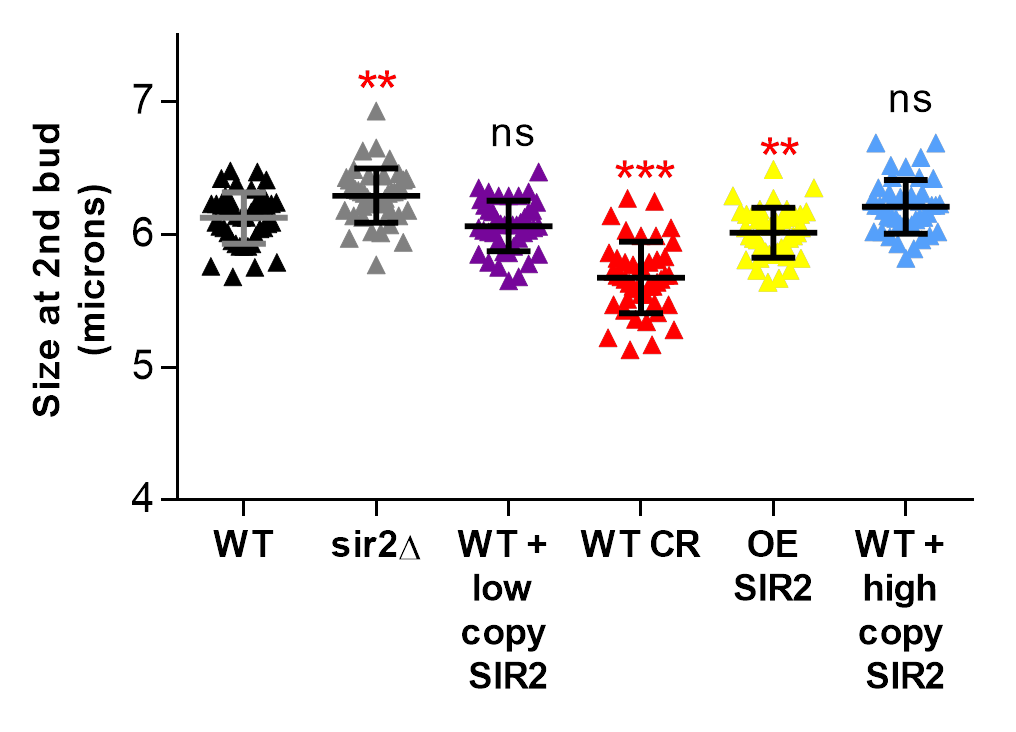

Supplement: S8 Fig — Wild type, sir2Δ, wild type transformed with a low copy SIR2 plasmid, wild type in CR, overexpression of SIR2 via an extra integrated copy of SIR2 (OE SIR2), and wild type transformed with a high copy SIR2 plasmid strains were imaged for several cell cycles in a Zeiss Axiovert microscope. The size of cells upon appearance of the second bud was measured. (** = p<0.001, *** = p<0.0001). (TIF) [file pone.0200275.s008.TIF]
